# Measles at the U.S.–Mexico border: The fragility of cross-border disease control

**DOI:** 10.1371/journal.pgph.0006207

**Published:** 2026-04-03

**Authors:** Enrique Chacon-Cruz, Katherine Wells, Janet Real-Ramírez, Tiffany Torres, Leonor Patricia Saltigeral-Simental, James Felberg, Erika Zoe Lopatynsky-Reyes

**Affiliations:** 1 Think Vaccines, LLC, Houston, Texas, United States of America; 2 Lubbock Public Health, Lubbock, Texas, United States of America; 3 Instituto Nacional de Enfermedades Respiratorias Ismael Cosio Villegas, Mexico City, Mexico; 4 Lubbock Public Health, Lubbock, Texas, United States of America; 5 Instituto Nacional de Pediatria, Mexico City, Mexico; 6 Texas Tech University Health Science Center School of Medicine, Lubbock, Texas, United States of America; 7 Think Vaccines, LLC, Houston, Texas, United States of America; PLOS: Public Library of Science, UNITED STATES OF AMERICA

Measles, a highly contagious, vaccine preventable disease, has re-emerged as a significant public health threat in North America, driven by intense cross-border mobility and persistent gaps in vaccination coverage. The U.S.–Mexico border operates as a single epidemiologic space, with millions of people crossing the border for work, family, commerce, and health care, allowing a highly contagious virus such as measles to spread easily in communities where immunization levels fall below the 95% two-dose threshold.

Recent surveillance highlights the scale of this resurgence: since 2025, the United States reported over 2,200 cases [1], while Mexico documented more than 9,000 cases and over 30 deaths [2].

**Initial cases and early transmission in Texas:**

Public health authorities were alerted to the West Texas measles outbreak on January 24, 2025, after a hospital in Lubbock reported suspected measles in two children from Gaines County. The outbreak was announced on January 29 [3].

Epidemiologic findings suggest broader community transmission, with reported illness clusters in unregistered private schools linked to Mennonite churches, including high absenteeism and temporary closures, though not independently verified.

The primary case in Mexico was an unvaccinated Mennonite child from Cuauhtémoc, Chihuahua, who traveled to Seminole, Gaines County, Texas (Jan 20–24, 2025), coinciding with early cases there. Fever began January 29, with laboratory confirmation on March 20 [4], supporting likely exposure in Texas.

This outbreak occurred in communities with less-than-optimal vaccination coverage. According to the Texas DSHS data for the 2024–2025 school year, MMR coverage in the county’s three school districts was 77% among kindergarteners and 85% among seventh graders, with particularly low coverage in Loop Independent School District, where only 20% of kindergarteners were vaccinated [5]. Meanwhile, in Mexico, the first dose of the MMR vaccine has only reached only 65% of children, while coverage for the second dose declined further to 50% [6].

In both states (Texas and Chihuahua), vaccination coverages were suboptimal, leaving a substantial proportion of the population susceptible to measles transmission. This led to the current outbreak, where to date, the US has reported over 3000 cases and three measles-related deaths [7], and Mexico has confirmed over 10,000 cases and 31 deaths [8]. The outbreak has spread widely in both countries.

The U.S.–Mexico border is one of the longest and busiest in the world, with over 350 million legal crossings annually [9]. In this context, continuous movement combined with pockets of under-immunization on both sides of the border creates ideal conditions for measles transmission, particularly in communities where vaccination coverage falls below the ~ 95% two-dose threshold needed to prevent sustained spread.

Why borders do not stop measles—and may amplify transmission:

Measles outbreaks occur when imported cases reach susceptible communities, expanding only where vaccination coverage is low. PAHO has repeatedly documented this pattern in the Americas, where immunity gaps allow sustained transmission [8]. Border regions often function as shared binational communities, with intertwined schools, workplaces, health care, and family networks, enabling rapid cross-border to spread. Importantly, localized pockets of under-immunization—rather than national vaccination averages—drive outbreaks, as seen in the current resurgence in both the United States and Mexico.

The U.S.–Mexico Border: a shared epidemiologic frontier:

The U.S.–Mexico Border Infectious Disease Surveillance (BIDS) program exists precisely because frequent cross-border movement facilitates the spread of communicable diseases, requiring coordinated binational surveillance and response. Here are the key components:

- Develop a collaborative framework for data sharing between U.S. and Mexican health authorities.
- Identify and monitor infectious disease trends in border communities.
- Implement joint training programs for healthcare professionals on disease detection and response.
- Utilize technology for real-time reporting and analysis of health data.
- Foster community engagement to raise awareness about infectious diseases.
- Establish rapid response teams for outbreaks that cross the border [9].

Measles is not the only pathogen causing cross-border outbreaks, but its extreme transmissibility makes it especially consequential. The history of binational outbreaks highlights the U.S.–Mexico border as a shared epidemiologic space where frequent movement and social interconnectedness facilitate disease spread.

Dengue illustrates this dynamic clearly, with outbreaks in northern Mexico repeatedly linked to cases in Texas border counties. Local transmission was documented in Brownsville in 2005 and again in 2013–2014 in several Texas counties, coinciding with outbreaks across the border [10]. Tuberculosis shows a similar pattern, with higher incidence in U.S. border counties, particularly among individuals born in Mexico, reflecting sustained cross-border mobility and health-system challenges [11]. Beyond single pathogens, binational studies report higher rates of enteric and respiratory infections—including vaccine-preventable diseases—in border communities, consistent with shared environments and frequent cross-border contact.

## Conclusions

Despite decades of progress, this outbreak exposes persistent structural vulnerabilities along the border, including under-vaccinated communities, uneven access to preventive services, and limited cross-national public health coordination. Measles thus serves as a sentinel event, highlighting broader weaknesses in cross-border preparedness. Preventing future outbreaks will require sustained binational strategies that ensure high two-dose vaccination coverage, implement targeted catch-up campaigns, and strengthen cross-border surveillance and response. Without coordinated action, the U.S.–Mexico border will remain a fault line for measles transmission and a warning for regional infectious disease control.

Examples of cross-border measles transmission are well documented [12]. In an era of intense human mobility, border regions function as epidemiologic corridors where heterogeneous vaccination coverage and uncertain immune status can rapidly facilitate viral spread. Strengthening vaccination strategies for migrants and mobile populations—together with robust catch-up campaigns to close immunity gaps—must therefore be a public health priority in all frontier settings. This is not only a disease-control measure, but also a matter of global health equity.

The urgency is further heightened by upcoming mass gatherings. In June 2026, Canada, the United States, and Mexico will jointly host the FIFA World Cup, bringing hundreds of thousands of international visitors to a highly interconnected trinational region. Such events amplify the risk of importation and exportation of measles and other vaccine-preventable diseases when immunity gaps persist.

Ensuring a well-vaccinated and epidemiologically coordinated border safeguards both neighboring countries and the global community. Sustained international commitment—including support for multilateral initiatives such as Gavi—is essential. Border health security is not just a national task, but it is also a shared global responsibility.
